# A Semi-Supervised Object Detector Based on Adaptive Weighted Active Learning and Orthogonal Data Augmentation

**DOI:** 10.3390/s25061798

**Published:** 2025-03-14

**Authors:** Meng Wang, Xiao Xu, Haipeng Liu

**Affiliations:** Faculty of Information Engineering and Automation, Kunming University of Science and Technology, Kunming 650500, China; 20222204219@stu.kust.edu.cn (X.X.);

**Keywords:** semi-supervised learning, active learning, contrastive learning, object detection, unlabeled data mining

## Abstract

To efficiently utilize limited resources, this paper proposes a semi-supervised object detection (SSOD) approach based on novel adaptive weighted active learning (AWAL) and orthogonal data augmentation (ODA). An uncertainty sampling framework is applied by adaptively weighting multiple evaluations to annotate the most informative samples for active learning. To further exploit the discriminant potential of unlabeled data, an adaptive weighted loss is introduced to fully mine the unlabeled data, and the normalized uncertainty score is adopted as the loss weight to explore low-score samples for training iterations. Moreover, an ODA operation is performed as pseudo-supervised learning on augmented instances to further capture the modality diversity of complex data distributions. Extensive evaluation and analysis are conducted on the MS-COCO dataset, achieving a mean average precision (mAP) of 35.10 with only 10% of the annotated data. Compared with the existing active learning baselines, the AWAL strategy improves the performance by 1.3% without the ODA. When ODA is incorporated, an additional performance gain of 1.2% is observed. Furthermore, training on the fully annotated MS-COCO with additional unlabeled data, the performance achieved at 43.30 mAP, demonstrating the superiority of the proposed approach.

## 1. Introduction

With the rapid advancement of deep learning technologies, the performance of computer vision tasks such as object detection [1,2,3,4,5,6] and image classification [7,8,9,10,11] has been significantly improved. Relying on large-scale annotated datasets [12,13,14,15] to supervise the training of model hyperparameters, the reasoning ability of object detectors is improving without a break. However, most naturally acquired images are unlabeled, and manual labeling is labor-intensive and costly. To this end, semi-supervised learning (SSL) [16,17] is often applied to alleviate the bottleneck of insufficient labeled data in practical scenarios. Recently, active learning (AL) [18,19,20,21,22,23,24,25,26,27] has also been introduced into the scenario of object detection, effectively optimizing sample learning strategies when label resources are scarce. In this paper, we focus on a novel AL strategy that can further capture the discriminant potential of unlabeled data and be applied to challenging scenarios of SSOD.

In recent years, SSOD [28,29,30,31,32,33,34,35,36] has become a hotspot in the field of computer vision. It aims to improve the learning performance of object detectors by using a few labeled samples and plenty of unlabeled samples. There are two main benchmarks for SSOD, and one is the pseudo-label-based approach [29,30,31,32,33,34,35], which is currently the most widely used for SSOD scenarios. Specifically, these models are based on teacher–student network (TSN) training that contains two object detectors, namely a teacher network (TN) and a student network (SN). The TN generates pseudo labels for unlabeled data with weak augmentation to supervise the training of the SN. This self-training scheme has been demonstrated to mitigate the challenge of insufficient labeled data. For example, the STAC proposed by Ksohn et al. [29], which is based on this scheme, greatly improved the performance of SSOD, fully demonstrating the effectiveness of the TSN and pseudo-labeling for SSOD. Many subsequent works have explored how to improve the quality of pseudo-labeling. For example, Instant Teaching [30] introduced a model ensemble to aggregate predictions from multiple teacher models to overcome the confirmation bias problem. Soft Teacher [32] applied a Soft Teacher mechanism to address the problem of low detection in pseudo-labels caused by high confidence thresholds. When calculating the student model training loss, the prediction score of the detection box predicted by the student model shared by the teacher model is used as a weight. In summary, the pseudo-label-based method has become the mainstream approach for SSOD.

Additionally, another benchmark suggested applying consistency constraints [33,34,35,36,37]. From the perspective of learning data modalities, these schemes are forced to have similar predictions with different data augmentation (DA) and perturbations after obtaining normal and perturbed inputs. For instance, CSD [37] is a typical SSOD method using consistency regularization. It flips the image horizontally and then feeds it into the network for training to produce constrained and consistent predictions with the non-flipped image. Later, some studies [31,32,33,34,35,36] combined the above two types of baselines to obtain the gains brought by each component. However, the quality of pseudo labels is generally inferior to that of true labels, and the contribution of unlabeled samples to model training is also uneven. Recently, by introducing the AL methodology to feed only high-quality unlabeled samples [26,27], the detector deterioration caused by low-quality samples during training can be further improved.

Different from traditional passive learning methodologies, AL aims to actively select data samples mostly suitable to be labeled, thereby improving learning performance while solving the problem of insufficient labeled data [26,27]. Typically, the most valuable samples for training can be selected by measuring the uncertainty, information gain, or diversity of these unlabeled samples. Recently, works [26,27,28] have applied AL to SSOD scenarios to enhance data utilization. Among them, Active Teacher [26] judges the detection difficulty of an image according to the predictions of the teacher model and then actively selects some samples for annotation to guide the model optimization [28]. The image stability is comprehensively judged by calculating its classification stability and positioning stability, and then images with low stability are selected for manual annotation to supervise the model learning. However, the above AL scheme only selects the most valuable samples to improve the efficiency of self-supervised training; therefore, most of the remaining low-scoring unlabeled samples are discarded. This leads to insufficient exploration of the patterns contained in these low-scoring samples and a lack of diversity in the training data. Therefore, further research is needed to develop learning strategies that effectively utilize previously discarded unlabeled data.

On the other hand, data augmentation (DA) has also been applied to improve the performance of SSOD. This DA solution is first introduced into the tasks of semi-supervised image classification [16,17]. Later, STAC [29] used DA to improve the SSOD baselines and achieved better results, and then subsequent methods [30,31,32,33,34,35,36] mostly consider DA as an indispensable part of the semi-supervised architecture. Typically, SSOD models process weakly augmented unlabeled samples through a teacher network (TN) to generate pseudo-labels while feeding strongly augmented versions of these samples into a student network (SN). This network is then trained using the pseudo-labels generated from the weakly augmented views. In general, the above DA strategies have the problem of generating a relatively simple distribution and causing a bottleneck of excessive regularization of training iterations due to the insufficient diversity of data patterns. Therefore, this paper attempts to expand efficient DA strategies further.

**Figure 1:**
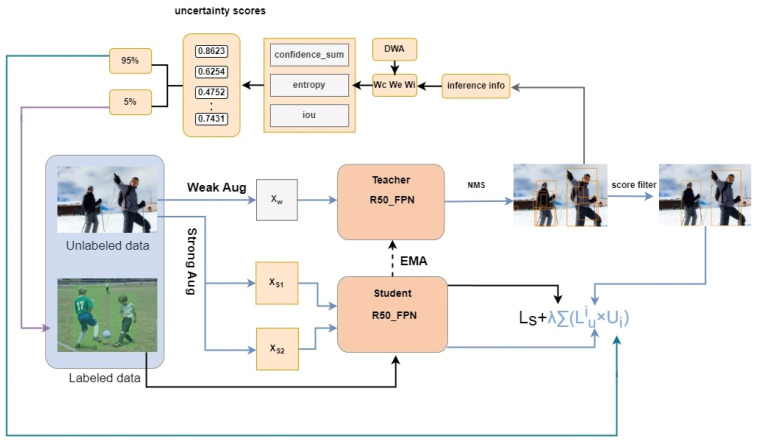
The overall framework of the AWAL and ODA solution proposed in this paper. AWAL is used to select the most valuable unlabeled samples for manual annotation and further mine unlabeled samples through loss weighting. In addition, the ODA in this paper is also applied to the training process to improve the robustness and generalization ability of the model. The loss consists of supervised loss and unsupervised loss.

To address the above challenges, this paper proposes an adaptive weighted active learning (AWAL) strategy and an orthogonal data augmentation (ODA) architecture, as shown in Figure 1. We focus on alleviating the problems of insufficient utilization of unlabeled samples and diversity of data augmentation mode.

To enhance the utilization of unlabeled data, this paper uses an AWAL strategy to integrate three indicators: trust, classification entropy, and bounding box to derive an uncertainty score for detection tasks. To this end, a sub-network that dynamically adjusts indicator weights based on model performance distribution is designed. By adaptively focusing on the emphasis indicators of the current sample for the detection task, the uncertainty measurement score provides more reliable and meaningful guidance. Additionally, in order to further improve the utilization of samples, in addition to selecting a small number of high-scoring samples for manual labeling, this paper uses the normalized uncertainty metric score as the training weight of the unsupervised loss of the remaining unlabeled samples. This approach ensures that low-scoring unlabeled samples are effectively utilized, thereby enhancing the overall model training process.

In addition, to address the lack of sample diversity caused by excessive iterative regularization in existing DA methods, an ODA architecture is applied based on a contrastive learning framework. Specifically, an additional branch of strong data augmentation (SDA) is introduced, which is approximately orthogonal to the original SDA branch for the SN. The pseudo-labels generated by the weakly augmented views from the TN are used to supervise both SDA branches. This enables the student network to learn richer detection patterns, thereby enhancing its robustness and generalization ability.

Experimental results on the benchmark MS-COCO show the superiority of the proposed approach compared with the existing active learning baselines. In general, the contributions of this paper can be summarized into the following aspects:

- An AWAL strategy is applied using an uncertainty sampling framework, which utilizes an uncertainty sampling framework to integrate multiple uncertainty scores through a dynamic weight adjustment (DWA) mechanism. This approach enables the accurate selection of the most informative samples from unlabeled data for annotation in order to improve the learning efficiency of the SSOD baseline while reducing the annotation cost.
- A pattern mining of a full unlabeled data strategy is proposed based on an improved loss function by incorporating the normalized uncertainty score of unlabeled samples into the loss calculation and dynamically adjusting their loss weights during model optimization. Hence, the utilization efficiency of low-scoring unlabeled data and the training performance are significantly improved.
- Based on the idea of contrastive learning, an ODA strategy is performed, which can relieve the lack of training sample diversity under SSOD scenarios and is easy to expand to other SSOD baselines.
- The proposed approach breaks through the limitation of traditional active learning that only focuses on high-scoring samples and achieves the full participation of unlabeled data in training; it also provides a new solution of loss optimization for SSOD models.

## 2. Related Work

**Semi-supervised object detection (SSOD):** Common SSOD methods mainly include consistency-based and pseudo-label-based methods. The consistency-based method imposes consistency regularization constraints on input images to ensure consistent predictions across different transformations. CSD [37] is a typical SSOD method based on consistency regularization. It applies horizontal flipping to the input image and trains the network to produce consistent predictions between the flipped and non-flipped versions. This method can work on both one-stage and two-stage detectors. The pseudo-label-based method relies on a TSN framework, where the trained TN generates pseudo-labels for unlabeled data to supervise the training of the SN. In recent years, many pseudo-label-based methods have been applied to SSOD [29,30,31,32,33,34,35,36]. Unbiased Teacher [31] adopts a two-stage framework, which alleviates the overfitting problem by training the region proposal network (RPN) and RoI head with pseudo labels, solves the pseudo label bias problem, and improves the quality of pseudo labels by using exponential moving average (EMA) and focal loss. Cross Rectify [38] exploits the differences between detectors to identify self-errors and improves the quality of pseudo-labeling through a cross-correction mechanism. MUM [39] concatenates mixed input images and reconstructs them in feature space in an SSOD framework. Active Teacher [26] combines the concepts of SSL and AL, aiming to improve learning effects by utilizing limited labeled data and a large amount of unlabeled data. The above methods have contributed to the development of the field of SSOD, but they lack the mining and utilization of unlabeled data. This paper aims to study the mining and utilization of unlabeled data.

**Data augmentation (DA):** DA is crucial to improving model generalization and robustness, which is the first step in SSOD. The original paper, FixMatch [17], used DA for semi-supervised image classification and achieved good results. Later, many researchers also used this strategy for SSOD [29,30,31,32] and proved its usefulness. Therefore, to enhance the robustness of the model and make rational use of unlabeled data information, DA has become an indispensable part of the current SSOD algorithm. The core principle of DA is to apply consistency regularization to augmented data, ensuring the consistency of output predictions across different transformations.

**Active learning (AL):** AL is a method that fits perfectly with SSL. In the field of machine learning, AL is a strategy designed to reduce the reliance on labeled data while enhancing model performance. Traditional machine learning methods [3,4,5] typically require a large amount of labeled data for training, but in some cases, obtaining labeled data may be very expensive or difficult. Therefore, some researchers have combined AL and SSL to solve the problem of insufficient labeled data. Active Teacher [26] uses the trained teacher model to predict unlabeled data and judges the detection difficulty of unlabeled images based on the prediction results. It then actively selects some samples for labeling to guide the learning of the model. One of the methods [27] is to calculate the classification stability and positioning stability of an image to comprehensively judge the stability of an image, select images with low stability for manual annotation, and add them to the label set to supervise the model learning. Both of these methods focus on selecting samples with high detection values for manual annotation and supervised learning, thereby improving model performance. These contributions have significantly advanced the integration of SSL and AL.

**Comparative learning (CL):** In the field of machine learning, CL is a learning paradigm that aims to learn feature representations in data through comparison to improve model performance. In recent years, many studies [40,41,42,43,44] have combined object detection with CL. DetcO [40] performs comparative learning between global images and local images to improve the detection ability of the model. FCSE [44] performs Few-Shot Object Detection via Contrastive Proposal Encoding (FSCE), which is a simple yet effective method for learning contrastive-aware object proposal encoding that helps classify detected objects. The core idea of CL is to learn data representations through comparison, ensuring that similar samples are closer in the representation space while dissimilar samples are farther apart. In CL, one or more pairs of samples are usually used for comparison, and the model is trained by minimizing the distance between similar samples and maximizing the distance between dissimilar samples. This approach enables the model to learn more robust and meaningful data representations during training, thereby enhancing its generalization ability. However, there are currently limited studies exploring the integration of CL with semi-supervised object detection (SSOD).

## 3. The Proposed AWAL-ODA Approach

This section will focus on the AWAL strategy and the ODA module based on CL. Specifically, the baseline framework adopted by the semi-supervised detection network in this paper will be introduced in Section 3.1, and the AWAL strategy and ODA module proposed in this paper will be introduced in Section 3.2 and Section 3.3, respectively.

### 3.1. SSOD Framework

In this paper, the semi-supervised detection model framework adopted is based on the TSN, which mainly includes a TN and an SN. Both networks use the Faster-RCNN [3] baseline, and the internal backbone network uses Resnet50_FPN [45]. Among them, TN generates pseudo-labels by inferring unlabeled data, which are then used to supervise the training of the SN. To provide a clearer explanation of the model training process, the following definition is made.

Given a set of manually annotated datasets $D_{S}$ = {$X_{L}$, $Y_{L}$} and an unlabeled dataset $D_{U}$ = {$X_{U}$}, where *X* represents samples and *Y* represents annotations. The goal of SSOD is to use a small amount of labeled data and a large amount of unlabeled data to train the object detector to maximize its performance.

The entire training process includes two stages: supervised training and self-supervised training. In the first stage, samples in the labeled data $D_{S}$ are first input into SN through weak augmentation mapping and supervised by $Y_{L}$ for training. Here, the supervision loss consists of classification loss and bounding box regression loss:

(1) $$L_{sup}=\frac{1}{N_{l}}\sum_{i=1}^{N_{l}}\left(L_{cls}\left(x_{l}^{i},y_{cls}^{i}\right)+L_{reg}\left(x_{l}^{i},y_{reg}^{i}\right)\right),$$

Among them, $L_{sup}$ is the supervision loss, $N_{l}$ is the number of labeled data, $N_{i}$ represents the i-th labeled sample, and $L_{cls}$ and $L_{reg}$ represent the classification loss and regression loss, respectively. For Faster-RCNN, RPN loss is also included:

(2) $$\left\{\begin{array}{c} L_{cls}\left(x_{l}^{i},y_{cls}^{i}\right)=L_{cls}^{rpn}\left(x_{l}^{i},y_{cls}^{i}\right)+L_{cls}^{roi}\left(x_{l}^{i},y_{cls}^{i}\right),\\ L_{reg}\left(x_{l}^{i},y_{reg}^{i}\right)=L_{reg}^{rpn}\left(x_{l}^{i},y_{reg}^{i}\right)+L_{reg}^{roi}\left(x_{l}^{i},y_{reg}^{i}\right), \end{array}\right.$$

where $L_{cls}^{rpn}$ and $L_{cls}^{roi}$ represent the classification loss of the RPN and the RCNN parts, respectively. Similarly, $L_{reg}^{rpn}$ and $L_{reg}^{roi}$ represent the regression loss of the RPN and RCNN, respectively, and their formulas are as follows:

(3) $$\left\{\begin{array}{c} L_{\mathrm{reg}}\left(x_{l}^{i},y_{\mathrm{reg}\;}^{i}\right)=\sum_{i\in \{x,y,w,\;h\}}{\mathrm{smooth}}_{L_{1}}\left(t_{c}^{i}-y_{c}^{i}\right),\\ {\mathrm{smooth}}_{L_{1}}\left(x\right)=\left\{\begin{array}{cc} 0.5x^{2} & \;\mathrm{if}\;|x|<1\\ |x|-0.5 & \;\mathrm{otherwise}\; \end{array},\right. \end{array}\right.$$

where $t_{c}$ is the c-th coordinate of the output image X.

In practical scenarios, foreground-background class imbalance in training samples samples is an inherent problem that needs to be solved in object detection, especially in the semi-supervised task setting. Usually, a high score threshold τ is used to ensure the accuracy of pseudo labels. However, this approach often exacerbates the scarcity of pseudo-labeled samples and worsens the foreground-background imbalance. In addition, when labeled data are scarce overall, the training examples of certain specific foreground categories may be greatly limited, which makes the model tend to predict the class with the advantage of sample number, thus causing prediction bias. To alleviate these problems, this paper follows the approach of Unbiased Teacher [31] and employs focal loss [5] to mitigate the issue of class imbalance. The formula is as follows:

(4) $$\left\{\begin{array}{c} FP=-\alpha_{t}{\left(1-p_{t}\right)}^{\gamma }\log\left(p_{t}\right)\\ p_{t}=\left\{\begin{array}{cc} p, & \;\mathrm{if}\;y=1\\ 1-p, & \;\mathrm{otherwise}\; \end{array},\right. \end{array}\right.$$

and the $\alpha_{t}$ and γ parameters adopt the default settings in the original focal loss paper [5].

In the second stage, the unsupervised training adopted is mainly based on the self-learning methodology. First, the weakly augmented unlabeled data $X_{u}^{w}=A^{w}\left(X_{u}\right)$ ($A^{w}$ is a weak augmentation method) is sent to the trained TN for inference to generate pseudo labels. In order to retain high-quality pseudo labels, a series of post-processing is required. First, a large number of redundant prediction boxes are removed through NMS, and then a threshold τ is set to retain pseudo labels with relatively high confidence. Then, the strongly augmented unlabeled data $X_{u}^{s}=A^{s}\left(X_{u}\right)$ ($A^{s}$ is the strong augmentation method) is sent to SN for training. This part of the training is supervised by the pseudo labels generated by the above TN.

Through experiments, it is found that the high-scoring pseudo-labels retained by the threshold τ cannot guarantee the quality of the regression box. As performed in the Unbiased Teacher [31], calculating the bounding box regression loss on the unlabeled images will cause the training to not converge, so our unsupervised loss is

(5) $$L_{unsup}=\frac{1}{N_{u}}\sum_{i=1}^{N_{u}}L_{cls}\left(x_{u}^{i},{\hat{y}}_{cls}^{i}\right),$$

where $N_{u}$ is the number of unlabeled samples, $L_{cls}$ is the same as formula (2), and ${\hat{y}}_{\mathrm{cls}\;}^{i}$ is the pseudo label generated by the TN.

The entire network training includes supervised training and unsupervised training. The total loss is composed of supervised loss and unsupervised loss. The formula is as follows:

(6) $$L_{\mathrm{total}\;}=L_{s}+\lambda L_{u},$$

where λ is a parameter that controls the contribution of the unsupervised loss.

To avoid overfitting and class imbalance problems, we update the parameters of the TN from the SN through the exponential moving average (EMA). This method has been proven to be effective in many previous excellent SSOD works. The formula is as follows:

(7) $$\theta_{t}^{i}\leftarrow \alpha \theta_{t}^{i-1}+\left(1-\alpha \right)\theta_{s}^{i},$$

where $\theta_{t}$ and $\theta_{s}$ are the parameters of the TN and the SN, respectively, and *i* is the ^i^-th step of training. And α is a hyperparameter that determines the speed of parameter transfer and is usually close to 1.

### 3.2. Adaptive Weighted Active Learning

#### 3.2.1. Multi-Metric and Dynamic Weighting Strategy

In order to improve the ability of SSOD to select the most valuable samples from unlabeled data $D_{U}$, a dynamically adjusted active learning framework is proposed. The framework adopts the uncertainty-driven sampling strategy widely used in AL [26,27]. Specifically, the detection results of each unlabeled sample $X_{U}$ are evaluated through the trained model to calculate the uncertainty of the sample. To this end, three core indicators ($m_{conf}$, $m_{en}$, and $m_{IOU}$) are introduced in this paper to quantify the uncertainty of the detection results. Building on this foundation, a dynamic weight adjustment (DWA) strategy is proposed, allowing the weight of each metric to adapt dynamically based on sample characteristics. Therefore, the unlabeled data samples are sorted in descending order according to the uncertainty scores, and a portion of the samples with the highest scores are selected for manual labeling. Next, the specific implementation of the indicator calculation and DWA strategy will be introduced in detail.

Next, the specific implementation of indicator calculation and weight adjustment strategy will be introduced in detail.

First, the confidence level reflects the degree of confidence of the model in its predictions. It is necessary to calculate the sum of the confidence scores of each detected object (bounding box). This indicator is calculated as follows:

(8) $$m_{conf}=\frac{1}{N}\sum_{i=1}^{N}s_{i},$$

where $s_{i}$ is the confidence score of the *i*-th bounding box predicted by the TN, and *N* is the number of bounding boxes. Since the bounding boxes left at the end are those left after a series of post-processing (including NMS), the higher the confidence score, the more certain the model is that the detection result is correct. Since the bounding boxes left at the end are those left after a series of post-processing (including NMS), the higher the confidence score, the more certain the model is that the detection result is correct.

Secondly, uncertainty reflects the uncertainty of the model when making classifications. Generally, the larger the classification entropy of an image, the flatter the model’s prediction distribution (i.e., the model’s probability estimates for multiple categories are similar), and the higher the uncertainty. Then, samples with high classification entropy indicate that the model is more uncertain about these samples. This indicator calculates the classification entropy for each detected bounding box and then calculates the sum of the classification entropies of all bounding boxes. The calculation method is as follows:

(9) $$m_{en}=-\sum_{i=1}^{N}s_{i}\cdot \log s_{i},$$

where $s_{i}$ is the confidence score of the *i*-th bounding box predicted by the TN, and *N* is the number of bounding boxes.

Finally, the bounding box overlap (IoU) measures the degree of overlap between two bounding boxes. For each image, we compute the IoU between each pair of detected bounding boxes. Then, the average of these IoUs is calculated. A high IoU average value indicates that the detected bounding boxes overlap heavily with each other, which may mean that the model is not localizing well enough on that image.

For each pair of bounding boxes, we first calculate their IoU, which is the ratio of the intersection area to the union area, as follows:

(10) $$\mathrm{IoU}\left(A,B\right)=\frac{\left|A\cap B\right|}{\left|A\cup B\right|},$$

Among them, *A* and *B* are two bounding boxes. On this basis, the average contribution of IOU of all bounding box pairs is calculated:

(11) $$m_{IOU}=\frac{1}{M}\sum_{i=1}^{N}\sum_{j=i+1}^{N}\mathrm{Io}U\left(A_{i},A_{j}\right),$$

where Io*U*($A_{i}$, $A_{j}$) is the IoU between the *i*-th and *j*-th bounding boxes, *N* is the number of bounding boxes, and *M* is the number of bounding box pairs.

**Figure 2:**
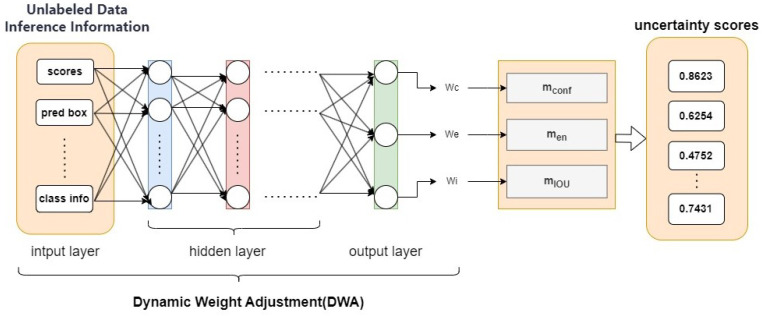
DWA based on model performance distribution. Each image has a different focus, and this solution can effectively concentrate learning resources on samples with poor performance.

To address the heterogeneous emphasis of uncertainty measurements across diverse image samples, this paper proposes a DWA Network based on model performance distribution to adaptively optimize uncertainty metric weights for different samples. The core insight lies in the recognition that a single uncertainty metric fails to comprehensively capture sample informativeness. For instance, while classification entropy reflects prediction uncertainty, it lacks sensitivity to spatial distribution characteristics (e.g., Intersection over Union, IOU). By integrating multiple metrics (including confidence scores, classification entropy, and IOU) with a dynamic weighting mechanism, our approach enables holistic uncertainty assessment. This multi-metric synthesis effectively models sample complexity, thereby enhancing sample selection precision. As illustrated in Figure 2, the DWA network processes inference information from the teacher model (TDN) for unlabeled samples (confidence, classification entropy, IOU, etc.), employs hidden layers to extract inter-feature relationships and patterns, thereby capturing category complexity and model prediction status. The network ultimately outputs a weight vector that dynamically modulates category-specific weights during training. This mechanism adaptively prioritizes categories with higher classification difficulty or lower confidence based on real-time model performance, thereby optimizing overall model efficacy through focused learning of critical categories.

The integrated loss function proposed in this study comprises two complementary components: category prediction loss and weighted loss. The category prediction loss quantifies prediction errors for each class through focal loss, while the weighted loss dynamically recalibrates class-specific contributions by incorporating adaptive weighting coefficients derived from model performance. This dual-component design empowers the network to autonomously optimize weight assignments through gradient-driven learning guided by real-time model behavior. Specifically, categories exhibiting lower confidence scores or higher error rates are automatically assigned elevated weighting values, thereby amplifying their influence during backpropagation to prioritize underperforming categories in the training process. Such loss formulation strategically directs optimization focus toward challenging samples, effectively enhancing the model’s discriminative capability on hard-to-classify categories through performance-aware gradient modulation and targeted parameter refinement.

**Category prediction loss:** the focal loss used in this paper is shown in Equation (4), which is used to measure the prediction error of the model for each category.

**Weighted loss:** The loss of each category is adjusted according to the weight of the category, reflecting the attention paid to different categories. For example, the weight of the category can be dynamically adjusted according to its classification entropy or error rate, and the weight of the low-confidence category is increased, and vice versa.

(12) $$L_{DAW}\;=\;w_{i}\cdot L_{class},$$

Among them, $w_{i}$ is the weight of each category predicted by the neural network, and $L_{class}$ is the loss of the corresponding category.

By dynamically adjusting the weights, the DWA network can focus more attention on categories or samples where the model performs poorly during training. This mechanism can accelerate the optimization of model performance, especially when the categories are unbalanced or some categories are difficult to classify.

Finally, the uncertainty score of the unlabeled sample is a comprehensive score after the weighted average of the three indicators, and its formula is as follows:

(13) $$v_{i}\;=\;w_{c}\cdot m_{conf}+w_{e}\cdot m_{en}+w_{i}\cdot m_{IOU},$$

Among them, $w_{c}$, $w_{e}$, $w_{i}$ are weight coefficients, which are optimized through the DWA network in Figure 2 as ($w_{c}$, $w_{e}$, $w_{i}$) = DWA (scores, pred box, …, class info) with trainable hidden layers. This allows weights to be calculated based on the current sample performance distribution to dynamically focus on different sample evaluation priorities.

The above three indicators can represent the uncertainty score of an image after adaptive weighted fusion. Compared with fixed weighting, the weights generated for us by the dynamically adjusted weight network based on the model performance distribution can better focus on the indicators emphasized by each unlabeled sample and generate a more valuable uncertainty score for the network.

#### 3.2.2. Pattern Mining for Full Unlabeled Data

In order to further explore the learnable modes of unlabeled samples, this paper proposes a novel sample loss calculation method to fully utilize the remaining unlabeled samples during training, thereby improving the learning efficiency of semi-supervised object detectors. According to the existing active learning framework, the training value of unlabeled samples is measured by calculating the uncertainty score. Theoretically, the higher the uncertainty score $v_{i}$, the greater the potential of the sample to improve model performance. However, previous studies [26,27] focused solely on selecting unlabeled samples with high uncertainty scores for manual annotation. This strategy has certain limitations in terms of optimization efficiency: although many low-scoring samples have small individual contributions, their information accumulation still has a significant effect on model training due to their large number, resulting in the problem of insufficient utilization of unlabeled data.

Based on the above analysis, this paper designs an improved sample loss function (Formula (16)) from a new perspective, aiming to directly integrate the uncertainty score of unlabeled samples into the loss calculation. The core idea is that no matter how high or low the uncertainty score is, every unlabeled sample has potential value in training. By adjusting the sample loss weight $w_{i}$ during training, even samples with low uncertainty scores can have a positive impact on model learning. This method can make full use of the potential contribution of the remaining low-scoring samples, thereby realizing pattern mining of full unlabeled data and significantly improving training efficiency and model performance.

(14) $$L_{u}\;=\;\sum_{i=1}^{N}L_{u}^{i}\cdot w_{i},$$

Among them, $L_{u}^{i}$ is the unsupervised loss of each unlabeled image, *N* is the number of images in the unlabeled data, and $w_{i}$ is the weight assigned to each unlabeled image.

The core of the method proposed in this paper is to optimize the loss function so that the uncertainty score of unlabeled samples is involved in the calculation of the total loss. During the training process, the unlabeled data are first predicted by the existing model, and the uncertainty score of each sample is calculated. Specifically, the calculation of the uncertainty score is consistent with the above method. Combining indicators such as confidence, classification entropy, and bounding box overlap (as shown in Formulas (10)–(13)), an uncertainty score $v_{i}$ is calculated for each unlabeled sample using Formula (15). This score is also the weight value assigned to the loss. For ease of calculation, we use the sigmoid activation function to normalize the uncertainty score to between 0 and 1.

(15) $$U_{i}=\frac{1}{1+e^{-v_{i}}},$$

where $v_{i}$ is a set containing the uncertainty scores of unlabeled samples.

In this way, the uncertainty score is normalized into a weight between 0 and 1. Based on this, an improved total loss function of the SSOD network is proposed:

(16) $$L_{total}=L_{S}+\lambda \sum_{i=1}^{N}L_{u}^{i}\cdot U_{i},$$

where $L_{S}$ is the supervised loss of labeled data, $L_{u}^{i}$ is the unsupervised loss of each unlabeled image, *N* is the number of unlabeled data images, $U_{i}$ is the weight obtained by normalizing the detection value score of each unlabeled image, and λ is an adjustment parameter used to balance the contribution of labeled data and unlabeled data to the total loss.

The improved loss function explicitly links the detection value of unlabeled samples with their loss by introducing the weight factor $U_{i}$. High-uncertainty samples will contribute more to the total loss and thus receive more attention, while low-uncertainty samples will also participate in model training with a smaller weight, thus ensuring the full utilization of unlabeled data.

### 3.3. Orthogonal Data Augmentation

**Figure 3:**
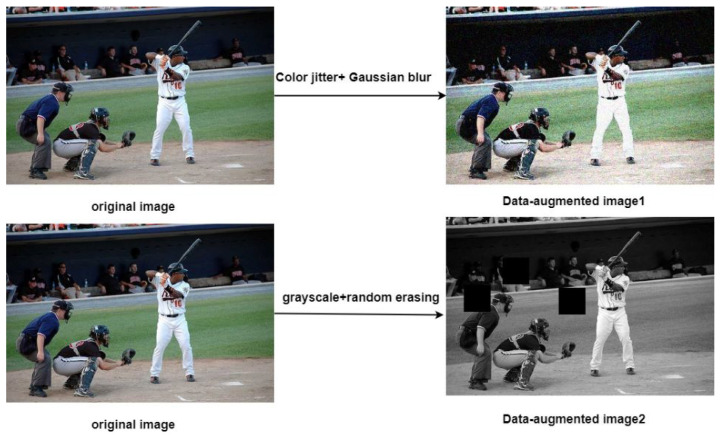
Orthogonal data augmentation. Color jitter+Gaussian blur and grayscale+random erasing in the figure are two different data augmentations, and their effects are independent and complementary; that is, they are orthogonal.

In mathematics and engineering, orthogonality usually refers to two or more vectors, functions, or operations that are independent in a sense; that is, their interactions are minimized. In the context of image data augmentation (DA), orthogonality refers to the fact that two or more DA strategies have independent and complementary effects when changing the input data. For example, the two DA strategies in Figure 3 are independent and complementary; that is, they are orthogonal. In other words, orthogonal data augmentation strategies do not repeat the same transformation pattern but perturb the data from different angles to generate diverse samples.

Object detection tasks face complex distributions of data, so applying different DA strategies can help the model learn more detection data patterns, thereby improving the model’s generalization ability and robustness. This paper is inspired by the idea of CL and combines it with DA. Specifically, for the same weak view $X_{w}$, we apply different strong augmentation strategies to generate two strong augmented views, $X_{s1}$ and $X_{s2}$, and then feed these two strong augmented views into the SN for training at the same time, using the pseudo-label $P_{w}$ from the same weak view for supervision. In addition, using the shared weak view $P_{w}$ to regularize the consistency of the two strong views, $X_{s1}$ and $X_{s2}$, can also be regarded as enhancing the consistency between them. This orthogonal combination of DA strategies not only helps the model learn more diverse data representations in different situations but also significantly improves the performance of the model, which is superior to the effect of a single augmentation stream.

In essence, this approach combines the advantages of CL and DA. By applying mutual ODA to the same image and using pseudo-labels from the same weak view for supervision, we are actually minimizing the distance between two similar samples, which is the core idea of CL. The core of CL lies in the contrastive loss function. By applying a contrastive learning loss similar to the InfoNCE loss, it can be seen that the model in this paper essentially has the similarity loss of Formula (17). Through the ODA strategy, the similarity of the same image under different augmented views is augmented, thereby improving the generalization ability and robustness of the model.

(17) $$L_{S1↔S2}=-\log\frac{\exp(\mathrm{sim}(S1,S2))/\tau }{\sum_{i=0}^{N}\exp\left(\mathrm{sim}\left(S_{i},K_{j}\right))/\tau \right.},$$

where *S*1 and *S*2 are positive sample pairs, usually different augmented views of the same unlabeled image with orthogonal augmentation methods; $S_{i}$ is the augmented view of the unlabeled image; $K_{j}$ is all comparison samples (including positive and negative samples); sim(a,b) represents the similarity between embeddings, usually cosine similarity; τ is the temperature parameter used to control the smoothness of the distribution; *N* is the number of mini-batch samples.

**Figure 4:**
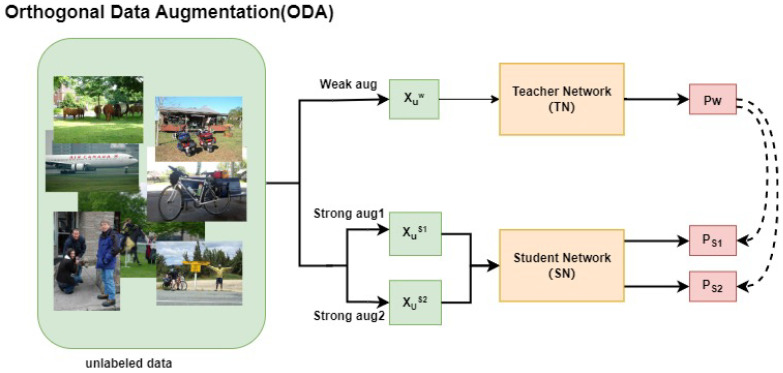
ODA module. Two orthogonal data augmentation samples are fed into the SN and supervised by the same pseudo label.

Figure 4 is the ODA module designed in this paper. First, the unlabeled data $X_{u}^{w}$ with weak augmentation is input into the TN for inference to generate the pseudo label $P_{w}$. Next, the weak view $X_{u}^{w}$ is sent to two different strong perturbation pools for augmentation to obtain $X_{u}^{s1}$ and $X_{u}^{s2}$. Subsequently, the two strongly augmented views are sent to the SN for training to generate predictions $P_{S1}$ and $P_{S2}$ and supervised by the same pseudo label $P_{w}$.

(18) $$\left\{\begin{array}{c} P_{w}=T\left(A^{w}\left(X_{u}\right)\right),\\ P_{S1}=S\left(A^{s1}\left(A^{w}\left(X_{u}\right)\right)\right),\\ P_{S2}=S\left(A^{s2}\left(A^{w}\left(X_{u}\right)\right)\right), \end{array}\right.$$

Among them, the teacher model T generates pseudo labels PW on weakly augmented images, while the student model S uses strongly augmented images for model optimization. $A^{s1}$ and $A^{s2}$ are two orthogonal SDA methods.

After integrating SSL and ODA, the total unsupervised loss is designed as follows: It is obtained by weighting the losses of the two SDA streams,

(19) $$L_{u}=\frac{1}{B_{u}}\sum ⊮\left(\max\left(p_{w}\right)\geq \tau \right)\cdot \frac{\mu }{2}\left(C\left(p_{w},P_{s1}\right)+C\left(P_{w},P_{s2}\right),\right.$$

where C represents a focal loss.

In summary, compared with the single-stream DA of most previous SSOD networks [29,30,31,32,33,34,35,36], the ODA in this paper learns more detection patterns. Moreover, under the influence of CL, the consistency regularization effect of ODA is better than that of single-stream data augmentation, and it contributes more to improving the generalization ability and robustness of the model.

## 4. Results and Discussion

### 4.1. Experimental Setup

**Datasets and evaluation:** We train and evaluate our method on two standard object detection datasets: MS-COCO [14] and PASCAL VOC [13]. The COCO dataset uses COCO train2017 and COCO unlabeled train. COCO train2017 contains 118k images, and COCO unlabeled train contains 123k images. VOC2007 trainval contains 5011 images, and VOC2012 trainval contains 16,551 images. Specifically, three experimental settings are used as follows:

1. COCO-partial: following (Sohn et al., 2020b) [29], we randomly extract 1%, 2%, 5%, and 10% of the images from the COCO train2017 set as the labeled set and use the remaining images in train2017 as the unlabeled set;
2. COCO-full: use the COCO train2017 set containing 118k images as the labeled set and an additional 123k unlabeled images as the unlabeled set;
3. PASCAL VOC: use the VOC07 training validation set as the labeled set, and use the VOC12 training validation set as the unlabeled set;

For evaluation, our method is evaluated using the COCO val2017 set and the PASCAL VOC07 test set.

**Experimental details.** Since existing SSOD methods [26,46,47,48,49,50] use a variety of different settings for training and testing, we evaluate our method under multiple settings for fair comparison. In all settings, the teacher model is first trained on the labeled set, and the student model is trained on a combination of labeled and unlabeled images. This paper reports the mean average precision (mAP) at different IoU thresholds (e.g., AP50, AP75, and AP50:95, denoted by AP) to measure effectiveness. Our experiments follow existing methods for fair comparison and thus use Faster-RCNN [3] as our detector and Resnet50_FPN (He et al., 2016) [45] as its backbone network. The optimizer we use is SGD, and the total number of training iterations is 300k. The learning rate is linearly increased from 0.001 to 0.01 in the first 1000 iterations and divided by 10 at iterations 299,990 and 299,995, respectively. Similar to [31], we use random horizontal flipping as the weak augmentation for TN learning, and the strong augmentations of the SN include color jitter, Gaussian blur, grayscale, and random erasing. We use a threshold τ = 0.7 to filter out low-quality pseudo labels. We use α = 0.9996 for EMA and λ = 2 for the unsupervised loss for all experiments. The batch size is set to sixteen; that is, eight labeled images and eight unlabeled images are obtained by random sampling.

### 4.2. Comparison Results and Analysis

In this section, the proposed method is compared with the existing state-of-the-art methods on MS-COCO. First, we evaluate our supervised baseline on a subset of labeled datasets and compare it to the Active Teacher [26] approach. In the benchmark, the results of the supervised baseline are similar to those reported by Active Teacher [26], as shown in Table 1. On this basis, the two methods are further compared at the system level, which is the advanced achievement in the field of AL and SSOD. Since this comparison focuses on AL methods, ODA strategies are not included in the experiments. Specifically, when the labeled data are 1%, 2%, 5%, 10%, and 20%, respectively, the proposed method outperforms Active Teacher [26] by 1.3 points, 1.2 points, 1.3 points, 1.3 points, and 1.1 points, respectively.

**COCO-PARTIAL results:** In Table 2, we compare the proposed semi-supervised detectors on COCO-PARTIAL with those in recent years, including CSD [37], STAC [29], Instant Teaching [30], Humble Teacher [34], Soft Teacher [32], Unbiased Teacher [31], Unbiased Teacher V2 [33], ACRST [51], and Active Teacher [26].

As can be seen from Table 2, the model performance of this paper is the best under the experimental settings with an annotated data ratio of 5% and 10%. When the annotated data ratio is 1% and 2%, the experimental results of this paper are only slightly worse than ACRST [51]. The specific reason may be that under extremely low annotated data ratios (1% and 2%), the category distribution in the annotated data is usually relatively unbalanced, and the samples of the minority category may be very scarce. The adaptive category rebalancing mechanism of ACRST [51] can adjust the category weights so that the model pays more attention to the samples of the minority category during training, thereby effectively alleviating the category imbalance problem. However, when the annotated data ratio is high (5% and 10%), the category distribution in the annotated data may tend to be balanced, and the category imbalance problem is no longer so prominent. At this time, the role of the adaptive category rebalancing mechanism of ACRST [51] will also be relatively weakened, and even unnecessary deviations will be introduced, resulting in a decrease in its performance gain. In contrast, compared with the baseline model, our model achieved continuous and stable improvements of 15.09, 14.59, 13.25, and 10.85 points in all evaluation data ratios, showing stronger robustness.

**VOC-PARTIAL results:** To fully verify the effectiveness and generalization ability of our method, we built a semi-supervised object detection experimental framework on the PASCAL VOC0712 dataset, using 5011 annotated images from VOC07 as labeled training sets and 11,540 unlabeled images from VOC12 as unlabeled data. Finally, we evaluated the method on 4952 test images from VOC07. The experimental results in Table 3 show that our method significantly outperforms the compared semi-supervised detection models with a mean average precision (mAP) of 51.31% and achieves an absolute performance improvement of 6.64%, 2.62%, and 1.72% over STAC [29] (44.64%), Unbiased Teacher [31] (48.69%), and ISMT [47] (49.59%), respectively. It is 9.18% higher than the supervised baseline (42.13%), highlighting its robustness under low-label data.

**COCO-addition results:** In order to further explore the performance gain potential of the fully supervised model after introducing unlabeled data, this paper constructed a semi-supervised extension experiment based on the MS COCO dataset. The experiment used 118k annotated images from COCO train2017 as the labeled dataset, 123k unlabeled images as the unlabeled dataset, and 5k images from COCO val2017 for rigorous testing. The results in Table 4 show that after 540k iterations of training, the model achieved significant improvement in the average precision (AP) of target detection: the overall AP continued to increase from 40.2% of the fully supervised model to 43.3% (+3.1%). Comparative experiments show that this method significantly outperforms mainstream semi-supervised strategies such as Unbiased Teacher [31] (+1.1%) and ACRST [51] (+2.59%). These results show that even when the fully supervised model has been fully trained, the performance bottleneck can still be broken through by effectively utilizing unlabeled data, verifying the significant advantages of this method in data utilization efficiency and model generalization ability.

### 4.3. Ablation Evaluations

In this section, the effectiveness of the design in this paper will be verified from two aspects: AWAL and ODA.

**Figure 5:**
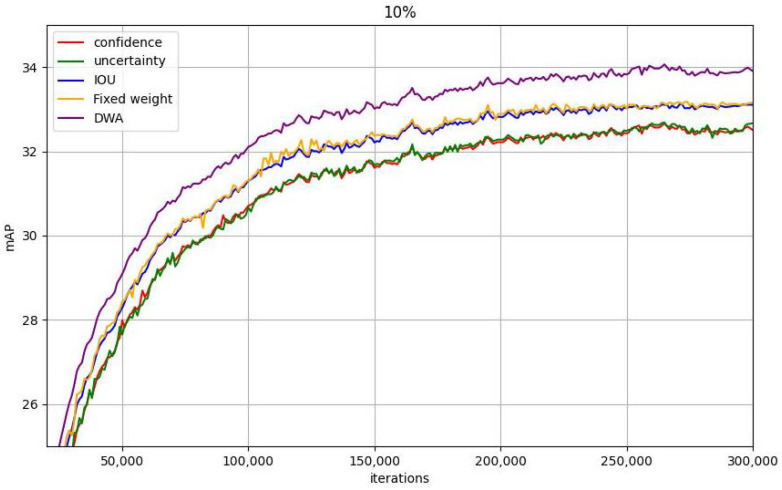
AP curves of different sampling indicators on 10% labeled data. The proposed (DWA) based on performance distribution can further combine the advantages of the three indicators.

**Adaptive weighted active learning:** First, we validated the effectiveness of the proposed metrics. From Table 5, it can be observed that the $m_{conf}$, $m_{en}$, and $m_{IOU}$ designed in this paper are all effective for semi-supervised detection. Moreover, the newly designed bounding box overlap rate can well help the detector capture samples with ambiguous positioning, bringing considerable performance gains to the detector. Figure 5 illustrates that the weight adjustment strategy based on the model performance distribution designed in this paper achieves higher gains compared with fixed weights. For categories with poor performance, the model can obtain more complex samples by increasing uncertainty ($m_{en}$) weights, thereby improving the performance of the model on these categories. For categories that already perform well, the model can further improve the generalization ability of the category by focusing on confidence ($m_{conf}$) and IOU ($m_{IOU}$). This method can focus on samples with fewer categories and poorer performance in the dataset. By dynamically adjusting the weights, the model can pay more attention to these categories, thereby gradually reducing the imbalance of categories. Secondly, as the training progresses, the model can gradually reduce its attention to simple samples and instead optimize more difficult categories. Table 6 shows that the proposed strategy of incorporating the uncertainty score as a weight into the unsupervised loss to enhance its contribution to model training can bring a gain of about 0.7AP. This shows that all unlabeled samples are beneficial to model training and should not be discarded. This paper finds a way to effectively utilize them without time-consuming manual labeling.

**Orthogonal data augmentation:** As shown in Table 2, the proposed ODA framework further improves the network performance, and different data DA combinations have varying effects on network performance. In the experiment shown in Table 2, the DA used in the first data stream is cutout and grayscale, and the DA used in the second data stream is color jitter and Gaussian noise. These two orthogonal data augmentation streams can allow the model to learn more detection representations and increase the robustness and generalization ability of the model. As shown in Table 7, this paper uses four data augmentations, namely random erasing, grayscale, color jitter, and Gaussian blur, for experiments. Different DA combinations bring different performance gains. Furthermore, Table 8 applies ODA to the STAC framework, resulting in additional performance gains for STAC and further validating the effectiveness of ODA.

### 4.4. Discussion of Visualization Results

**Figure 6:**
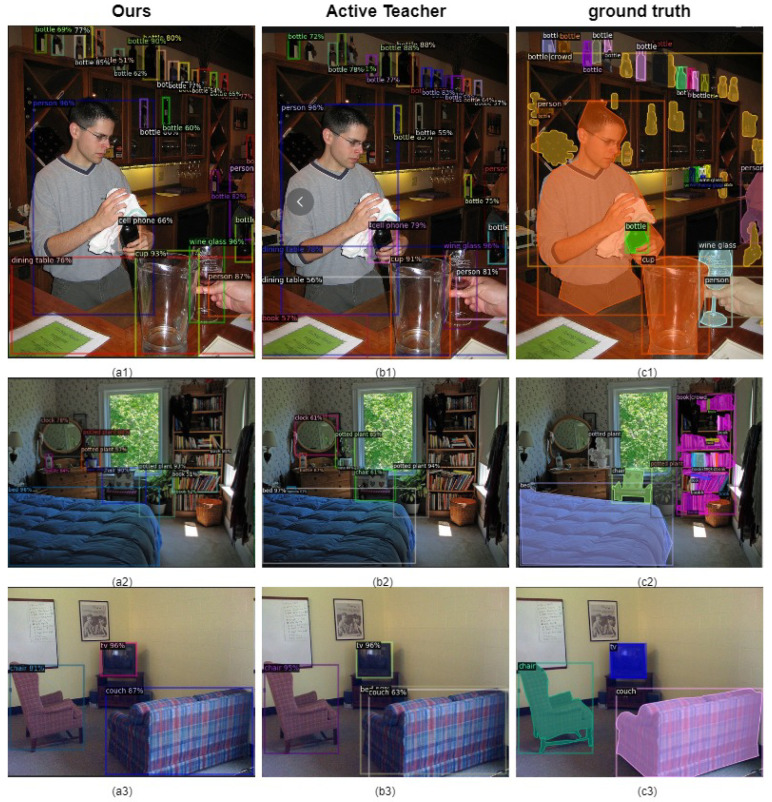
Visualization results. Comparison of the visualization effects of our model and the Active Teacher model with 10% labeled data samples. (**a1**–**a3**) are graphs of the results of our model detection, (**b1**–**b3**) are graphs of the results of the Active Teacher network detection, and (**c1**–**c3**) are manually labeled.

To further illustrate the effectiveness of our model, we visualize the prediction results and compare them with the Active Teacher [26] model. As shown in Figure 6, the prediction results of the proposed method and the Active Teacher [26] have high classification and positioning accuracy compared with the true annotations. However, upon closer inspection, the predictions of the proposed model are more accurately aligned with the ground truth annotations than those of the Active Teacher model [26]. We believe that the bounding box overlap rate metric newly designed in this paper is of great help to model positioning and can better locate samples with blurred bounding boxes. As shown in Figure 6, in Group 1 (a1, b1, c1), our model detects all the wine bottles at the top of the picture completely and accurately, and in Group 2 (a2, b2, c2), the books on the bookshelf are not all detected. However, compared with the Active Teacher [26], our model performs better. The sofas in the last group (a3, b3, c3) can be accurately detected by our model, while the Active Teacher [26] has fuzzy positioning. We believe this improvement is primarily due to the effectiveness of the bounding box overlap rate metric proposed in this paper.

## 5. Conclusions

This paper proposes a semi-supervised object detection method integrating an adaptive weighted active learning strategy and an orthogonal data augmentation strategy. The adaptive weighting strategy dynamically adjusts model performance metrics by incorporating uncertainty scores of unlabeled samples into the loss function, enabling comprehensive exploitation of unlabeled data while overcoming the limitations of conventional active learning approaches that prioritize high-confidence samples. The orthogonal data augmentation strategy, grounded in contrastive learning principles, enhances model robustness and generalization through diversified scenario modeling. Experimental results demonstrate significant performance improvements, though challenges persist, including potential weight prediction biases in scenarios with imbalanced data distributions and insufficient coverage of existing augmentation strategies for extreme lighting conditions, occlusions, and other complex scenarios. Future work will focus on optimizing category-sensitive weighting mechanisms and constructing a more comprehensive data augmentation framework to enhance the algorithm’s adaptability in real-world complex environments.

## Figures and Tables

**Table 1:** Detailed comparison of the performance of our network and the Active Teacher [26] network.

Method	1%	2%	5%	10%	20%
Supervised baseline (ours)	9.63	12.94	19.36	24.25	27.03
Supervised baseline (Active Teacher [26])	9.05	12.70	18.47	23.89	26.88
Active Teacher [26]	22.20	24.99	30.07	32.58	35.49
Ours	23.50	26.22	31.36	33.8	36.60

**Table 2:** Performance comparison of our network and other excellent semi-supervised detectors on COCO val2017. All networks use Resnet50_FPN [45] as backbone.

Method	1%	2%	5%	10%
Supervised	9.63	12.94	19.36	24.25
CSD [37]	10.51	13.93	18.63	22.46
STAC [29]	13.97	18.25	24.38	28.64
Instant Teaching [30]	18.05	22.45	26.75	30.40
ISMT [47]	18.88	22.43	26.37	30.53
MUM [39]	21.88	24.84	28.52	31.87
Humble Teacher [34]	16.96	21.72	27.70	31.61
Unbiased Teacher [31]	20.75	24.30	28.27	31.50
Unbiased TeacherV2 [33]	21.84	26.14	30.06	33.50
Soft Teacher [32]	20.46	-	30.74	34.04
ACRST [51]	26.07	28.69	31.35	34.92
Active Teacher [26]	22.20	24.99	30.07	32.58
Ours	24.72	27.53	32.61	35.10

**Table 3:** Comparison of some experimental results with other semi-supervised detectors using the VOC07 labeled dataset and the VOC12 unlabeled dataset.

Method	Labeled Set	Unlabeled Set	${\mathrm{AP}}_{75}$	${\mathrm{AP}}_{50:95}$
Labeled Only	VOC07	VOC12	72.63	42.13
CSD [37]	VOC07	VOC12	74.70	-
SATC [29]	VOC07	VOC12	77.45	44.64
Instant Teaching [30]	VOC07	VOC12	79.20	50.00
ISMT [47]	VOC07	VOC12	77.75	49.59
MUM [39]	VOC07	VOC12	78.94	50.22
Unbiased Teacher [31]	VOC07	VOC12	77.37	48.69
ACRST [51]	VOC07	VOC12	78.16	50.12
Ours	VOC07	VOC12	80.29	51.31

**Table 4:** Experimental results on COCO-additional.

Method	Iterations	${\mathrm{AP}}_{50:95}$
CSD [37]	270k	40.20→38.82
STAC [29]	540k	39.50→39.20
Unbiased Teacher [31]	270k	40.20→41.30
ACRST [51]	270k	40.20→42.79
Soft Teacher [32]	370k	40.90→44.50
Ours	540k	40.20→43.30

**Table 5:** Research on different sampling strategies for AL. In order to compare with the Active Teacher, this experiment did not add an ODA strategy, so the performance is different from that in Table 2.

Method	Metrics			COCO-PARTIAL	
	Confidence	Uncertainty	IOU	5%(2.5% + 2.5%)	10%(5% + 5%)
baseline				28.74	32.26
confidence	✓			29.81	32.50
uncertainty		✓		29.92	32.42
IOU			✓	30.60	33.12
Fixed weight	✓	✓	✓	30.67	33.16
DWA	✓	✓	✓	31.41	33.91

**Table 6:** Experiments on mining and utilizing selected samples. †: The uncertainty scores of the remaining unlabeled samples are added to the loss to increase their contribution to model training.

Method	5%	10%
Active Teacher [26]	30.07	32.58
Active Teacher †	30.78	33.31
Ours	31.93	34.45
Ours †	32.61	35.10

**Table 7:** Experimental results of mutually ODA under 5% and 10% labeled data. The following experimental results do not include active learning-related strategies, so the performance is different from Table 2.

s1 + s2	5%	10%
Color jitter, Gaussian blur + grayscale, random erasing	31.47	33.96
Grayscale, Gaussian blur + color jitter, random erasing	31.25	33.78
Random erasing, Gaussian blur + color jitter, grayscale	31.34	33.84

**Table 8:** Experimental results of adding ODA to STAC under 5% and 10% label data.

Method	5%	10%
STAC [29]	24.38	28.64
STAC + ODA	25.84	30.13

## Data Availability

Publicly available datasets were analyzed in this study. These data can be found here: MS-COCO: https://cocodataset.org/#download (accessed on 26 April 2024). PASCAL VOC: https://pjreddie.com/projects/pascal-voc-dataset-mirror/ (accessed on 26 April 2024).
